# AI post-intervention operational and functional outcomes prediction in ischemic stroke patients using MRIs

**DOI:** 10.1186/s12880-025-01864-1

**Published:** 2025-08-14

**Authors:** Emily Wittrup, John Reavey-Cantwell, Aditya S. Pandey, Dennis J. Rivet II, Kayvan Najarian

**Affiliations:** 1 Gilbert S. Omenn Computational Medicine and Bioinformatics, University of Michigan, Ann Arbor, MI USA; 2 Department of Neurosurgery, Virginia Commonwealth University, Richmond, VA USA; 3 Department of Neurosurgery, Michigan Medicine, Ann Arbor, MI USA; 4 Max Harry Weil Institute for Critical Care Research and Innovation, Michigan Medicine, Ann Arbor, MI USA; 5 Michigan Institute for Data and AI in Society (MIDAS), University of Michigan, Ann Arbor, MI USA; 6 Center for Data-Driven Drug Development and Treatment Assessment (DATA), University of Michigan, Ann Arbor, MI USA

**Keywords:** Acute ischemic stroke, Resnet, Autoencoder, Deep learning, Artificial intelligence, MRI, Modified rankin scale, Length of stay

## Abstract

**Background:**

Despite the potential clinical utility for acute ischemic stroke patients, predicting short-term operational outcomes like length of stay (LOS) and long-term functional outcomes such as the 90-day Modified Rankin Scale (mRS) remain a challenge, with limited current clinical guidance on expected patient trajectories. Machine learning approaches have increasingly aimed to bridge this gap, often utilizing admission-based clinical features; yet, the integration of imaging biomarkers remains underexplored, especially regarding whole 2.5D image fusion using advanced deep learning techniques.

**Methods:**

This study introduces a novel method leveraging autoencoders to integrate 2.5D diffusion weighted imaging (DWI) with clinical features for refined outcome prediction.

**Results:**

Results on a comprehensive dataset of AIS patients demonstrate that our autoencoder-based method has comparable performance to traditional convolutional neural networks image fusion methods and clinical data alone (LOS $>$ 8 days: AUC 0.817, AUPRC 0.573, F1-Score 0.552; 90-day mRS $>$ 2: AUC 0.754, AUPRC 0.685, F1-Score 0.626).

**Conclusion:**

This novel integration of imaging and clinical data for post-intervention stroke prognosis has numerous computational and operational advantages over traditional image fusion methods. While further validation of the presented models is necessary before adoption, this approach aims to enhance personalized patient management and operational decision-making in healthcare settings.

**Clinical trial number:**

Not applicable.

**Supplementary Information:**

The online version contains supplementary material available at 10.1186/s12880-025-01864-1.

## Introduction

Approximately 700,000 acute ischemic strokes (AISs) occur every year in the United States [1, 2]. The pathological cause is a blockage in a blood vessel that restricts the delivery of oxygen to parts of the brain [3]. The core affected area is thought to experience damage within the first few minutes of blockage, but early reperfusion, or restoration of blood flow, has shown success in saving the surrounding region from additional damage [4]. The choice of reperfusion therapy is in part clinically informed by medical imaging such as computed tomography angiography (CTA) taken on admission as part of the standard of care for stroke patients. After the intervention is selected and performed, if necessary, a magnetic resonance image (MRI) is typically performed to assess the severity of damage to both the core affected area and surrounding region. Recovery trajectory and long term effects are inherently impacted by the location and size of the damaged region in the brain. However, there exists little clinical guidance as to expected short- and long-term outcomes for patients including length of stay and 3 month functional outcomes.

Previous work has attempted to utilize machine learning to predict both short-term operational outcomes [5–13] and long-term functional outcomes, namely 90-day Modified Rankin Scale (mRS) [6, 11, 12, 14, 14–31, 31–51] following AIS. The majority of studies have utilized clinical data exclusively that can be extracted from the electronic health records (EHRs) such as medical history and demographics. Some attempt to predict outcomes using only admission-based features [5, 25, 26, 34, 40, 52], while others incorporate or focus on specific interventions [10, 33, 39, 43, 53]. For example, Zihni et al. compared different machine learning methods to predict long-term functional outcomes for stroke patients using EHR, initial stroke severity, and type of reperfusion intervention [53].

Given the inherent importance of the volume and location of injury in the brain, some studies have sought to incorporate imaging biomarkers. In the simplest case, they augment the EHR data with interpretable image derived features (IDF) such as lesion volume, thrombus location, or Alberta Stroke Program Early CT Score (ASPECTS) [10, 12, 13, 15, 18, 19, 21–23, 34, 39, 44–46]. For example, Yang et al. curated dynamic and static radiomics features from dynamic susceptibility contrast perfusion-weighted imaging (DSC-PWI) [28]. Alternatively, with the improvement of deep learning algorithms for image analysis, more studies have attempted more sophisticated methods of fusing the EHR and imaging data. Although many medical imaging problems can be simplified into a 2D framework which requires a smaller network, less parameters, and is easier to train, the pathology of AIS requires incorporation of the entire 2.5D image to fully characterize the injury. This has been attempted in the context of stroke outcomes with computed tomography (CT) [9, 11, 24, 26, 42, 52], CTA [25, 33], CT perfusion [9, 32], and MRI [17, 20, 27, 38, 54]. Other than White et al. which proposed a method that attempted to convert the 2.5D MRI into a 2D problem to use conventional networks [55], these studies overwhelmingly utilize 2.5D convolutional neural networks (CNN), particularly pre-trained Resnet architectures. Studies such as Ramos et al. [33] utilize late fusion to combine the tabular information, such as EHR, with the imaging data in the network prior to the prediction layer. Alternatively, Borsos et al. recently developed an alternative to late fusion when combining imaging data with EHR for AIS outcome prediction, dubbed DAFT (Dynamic Affine Feature Map Transform) [32].

These previous studies have not consistently shown a significant improvement in predictive performance when adding the 2.5D imaging to IDF and EHR. Furthermore, the CNN architecture is rigid, making it difficult to incorporate other imaging modalities, generalize to other outcomes, or support more advanced analysis. Therefore, the contributions of this work are as follows:

- Propose a new method of 2.5D MRI fusion with EHR using autoencoders for enhanced prediction of AIS outcomes.
- Apply existing and new methods for short and long-term AIS outcome prediction on a large public dataset establishing a benchmark for future methods.
- Robustly compare the use of clinical, image, and IDF both individually and in combination for the prediction of AIS outcomes.
- Explore prediction of inpatient length of stay (LOS), an important short-term operational outcome which has not been studied as diligently as long-term functional outcomes.
- Discuss limitations of short- and long-term outcome predictions for AIS patients and potentially barriers for implementation.

The following sections will detail the data and methods utilized for this study (Sect. “Methods”), the results of the analysis (Sect. “Results”) , and compare the performance to previous work (Sect. “Discussion”).

## Methods

### Dataset

This study utilized an Inter-university Consortium for Political and Social Research (ICPSR) dataset titled “Annotated Clinical MRIs and Linked Metadata of Patients with Acute Stroke, Baltimore, Maryland, 2009–2019 (ICPSR 38,464)” [56]. This dataset contains $2888$ patients admitted from 2009 to 2019 to the Johns Hopkins Comprehensive Stroke Center with a clinical diagnosis of acute stroke or early subacute stroke [36]. This dataset includes a variety of EHR information including medical history, demographics, hospital admission evaluations, and whether an intervention was performed. In addition, it includes post-intervention clinical MRI sequences with manually segmented lesion masks. Of the $2888$ patients in this dataset, $2164$ had a clinical ischemic stroke diagnosis at admission. Additional information about the dataset can be found in the corresponding publication [36].

### Cohort selection

**Fig. 1:**
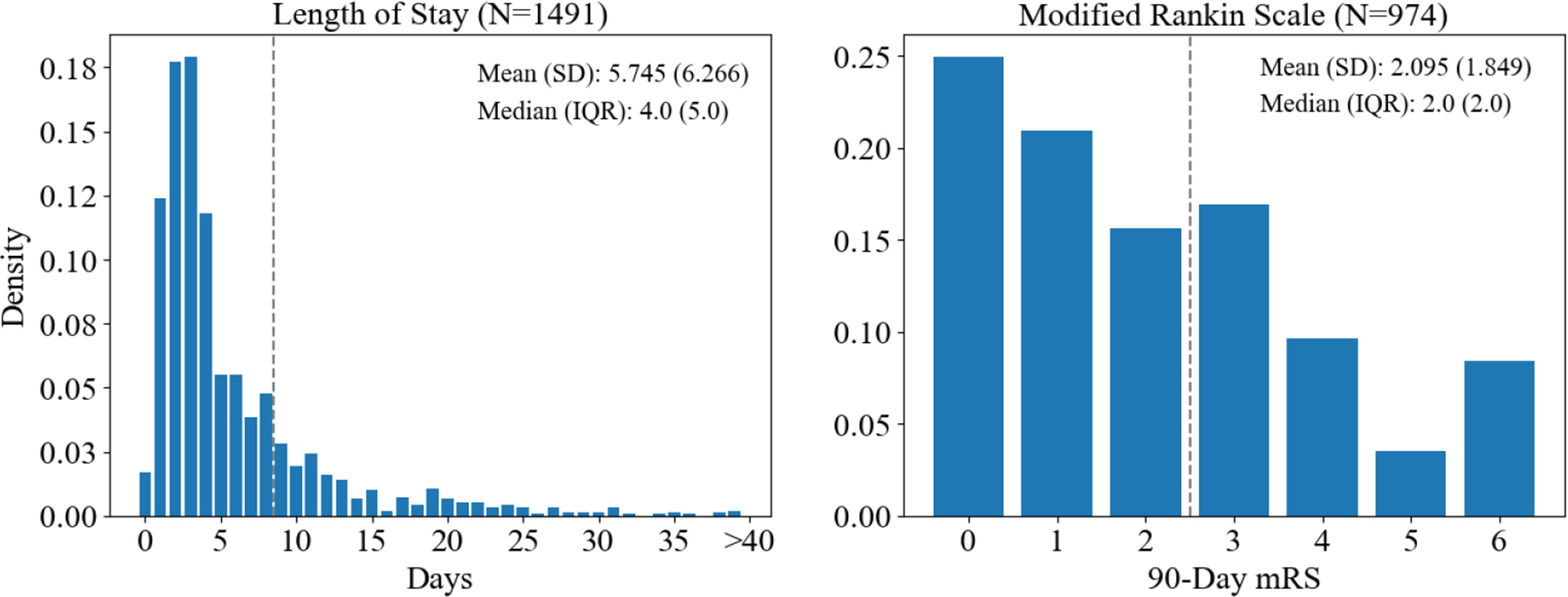
Distribution of outcome variables for each cohort presented as density histograms. Vertical gray dotted lines show the selected dichotomization thresholds. Three patients had a length of stay (LOS) $> 40$ days. SD: standard deviation; IQR: interquartile range; mRS: modified Rankin Scale

Two outcomes were selected for this study: LOS (short-term operational outcome) and 90-day mRS (long-term functional outcome). Modified Rankin Scale is a 7 point scale measuring disability for stroke patients with 0 representing no disability and 6 representing death. The distributions of these outcomes for AIS patients from the ICPSR dataset are shown in Fig. 1. These continuous variables were dichotomized to adapt the outcome measures for binary classification. Poor outcomes (positive samples) were defined as LOS $$ >$$ 8 days and 90-day mRS $$ >$$ 2 following the definitions proposed by previous studies for LOS [6] and mRS [11, 12, 26, 28, 32, 33, 39, 53] respectively. The third quartile of LOS in this dataset was 7 days, which is similar to the 8 day threshold established in literature. Furthermore, two on the mRS represents needing no assistance, making it a natural cutoff for functional outcomes.

Since some patients had missing outcome data, two cohorts were derived. Each outcome’s cohort contained the patients for which that outcome was available, meaning that the cohorts contain overlapping samples. The resulting cohorts had $1491$ and $974$ patients for LOS and 90-day mRS respectively. The demographic profiles of the overall AIS cohort in the ICPSR dataset in addition to the derived cohorts from outcome availability for LOS and 90-day mRS are presented in Table 1. To compare demographic distributions between the overlapping LOS and 90-day mRS cohorts, permutation tests (*n* = 1000) were employed to assess statistical significance. Furthermore, the demographic characteristics of the dichotomized groups are presented in Appendix A Tables A1 and A2 for the LOS and 90-day mRS cohorts respectively. The cohort for each outcome was randomly split into training (80%) and testing (20%) sets stratified by outcome.

**Table 1:** Demographic, stroke and imaging characteristics, and outcome occurrence for all ischemic stroke patients (total), ischemic stroke patients with available length of stay (LOS), and ischemic stroke patients with available 90-day modified Rankin Scale (mRS) respectively

	Total	LOS	90-day mRS	p^†^
	(*N* = 2164)	(*N* = 1491)	(*N* = 974)	
**Age** (years)	61.94 (14.28)	62.28 (14.05)	62.41 (13.65)	0.84
**Male**	1153 (53.28%)	803 (53.86%)	540 (55.44%)	0.45
**BMI** ($kg/m^2$)	29.23 (7.53)	29.28 (7.47)	29.59 (7.56)	0.35
**Race**				0.92
Asian	48 (2.22%)	40 (2.68%)	28 (2.88%)	
African American	971 (44.88%)	816 (54.73%)	526 (54.00%)	
Caucasian	607 (28.05%)	512 (34.34%)	336 (34.45%)	
*Missing*	538 (24.86%)	123 (8.25%)	84 (8.62%)	
**NIHSS Initial**	6.11 (6.57)	6.21 (6.65)	6.00 (6.69)	0.49
**IVtPA before scan**	1078 (49.82%)	1000 (67.07%)	729 (74.85%)	0.00*
**Lesion Volume** ($cm^3$)	24.24 (51.19)	25.19 (54.53)	24.60 (54.13)	0.81
**Thrombus Location**				0.66
Bilateral	279 (12.89%)	208 (13.95%)	122 (12.53%)	
Left	882 (40.76%)	594 (39.84%)	401 (41.17%)	
Right	799 (36.92%)	545 (36.55%)	364 (37.37%)	
*Missing*	204 (9.43%)	144 (9.66%)	87 (8.93%)	
**Scanner Manufacturer**				0.16
Siemens	1929 (89.14%)	1431 (95.98%)	947 (97.23%)	
GE	179 (8.27%)	48 (3.22%)	22 (2.26%)	
Phillips	19 (0.88%)	7 (0.47%)	3 (0.31%)	
Unknown	37 (1.71%)	5 (0.34%)	2 (0.21%)	
**Field Strength** (*T*)				0.23
1.5	1370 (63.31%)	908 (60.90%)	570 (58.52%)	
3.0	794 (36.69%)	583 (39.10%)	404 (41.48%)	
**LOS (days)**$\boldsymbol{ > 8}$	282 (13.03%)	282 (18.91%)	-	-
**90-day mRS**$\boldsymbol{ > 2}$	375 (17.33%)	-	375 (38.50%)	-

† p-values from permutation tests (*n* = 1000) between overlapping LOS and 90-day mRS cohorts

Values for age, BMI, NIHSS initial, and lesion volume are displayed as mean (standard deviations) while all other values are displayed as count (percentage). LOS: length of Stay; mRS: modified Rankin Scale; NIHSS: National Institutes of health stroke Scale total; BMI: Body Mass Index; IVtPA: thrombolysis with intravenous tissue-type plasminogen Activator

#### Tabular data

In addition to the features described in Table 1, the remainder of the tabular features included in modeling are listed in Table A3. Missing continuous variables in both the training and testing sets were imputed using the mean values calculated from the training set. In the training data, less than 20% per class of all continuous variables were missing. Subsequently, all continuous variables were standardized using StandardScaler [57] fit on the training set. Previous medications and previous medical conditions were provided as strings and were therefore one-hot encoded and assumed false if absent. This resulted in 40 clinical features and 2 IDF: thrombus location and lesion volume. The latter were both curated and provided as part of the ICPSR dataset.

#### Imaging data

The ICPSR dataset included numerous clinical MRI sequences including diffusion weighted imaging (DWI) which was selected for this study [58]. The images, provided as a sequence of 2D slices, were taken with at least 3 different scanners and two different field strengths as shown in Table 1. Additional information on the sequence parameters can be found in the dataset documentation [56]. The 2.5D volumes were mapped to Montreal Neurological Institute (MNI) space and intensity normalized prior to publication on ICPSR [36]. In addition, skull stripping was performed to create brain masks and manual segmentation was performed to create stroke masks [36].

### Modeling architectures

**Fig. 2:**
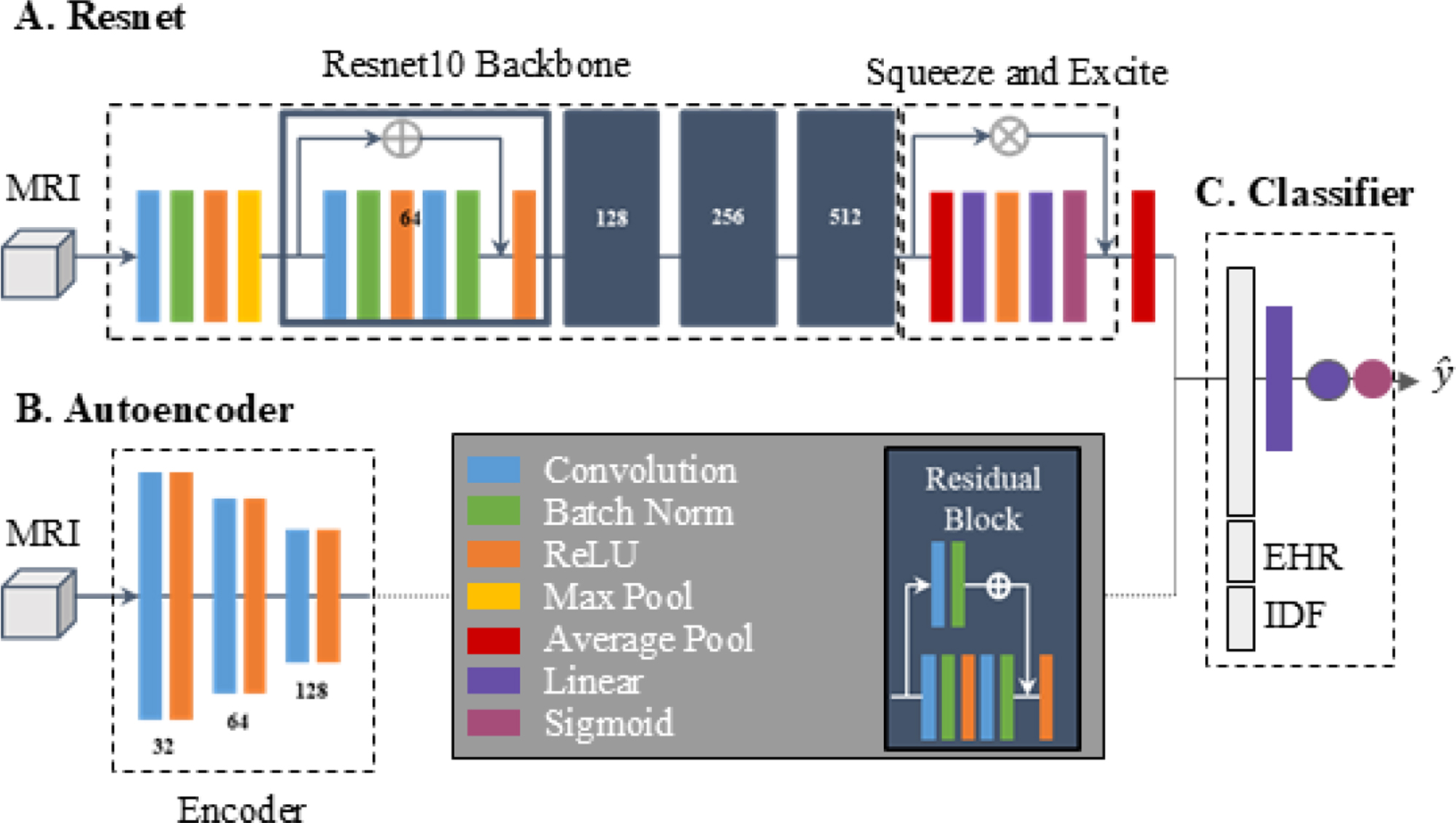
Architecture diagram of **A**. Resnet10, **B**. Autoencoder encoder, and **C**. Classifier. MRI: magnetic resonance imaging; EHR: electronic health records; IDF: image-derived features; ReLU: Rectified; avg: average

A diagram of all utilized architectures is presented in Fig. 2 and a detailed description of each is provided in Appendix B. In brief, a single layer neural network architecture is utilized for classification and two methods of deep learning are employed to extract information from the 2.5D MRIs: Resnet and autoencoders.

### Experimental setup

The training set for each cohort was shuffled into three stratified folds of 80% training and 20% validation using scikit-learn’s StratifiedShuffleSplit function [57]. The hold-out test sets mentioned in Sect. “Cohort Selection” for each cohort were untouched during training and identical across all folds. The same train/validation folds were preserved across all experiments and performance metrics were averaged across the three folds. The area under the precision recall curve (AUPRC) was the primary metric used to compare model performance given the class imbalance in the dataset due to the rarity of the severe outcomes. In addition F1-Score was used to add context on precision and recall. Additional experimental setup details are provided in Appendix C.

Three types of experiments were performed using different types and combinations of data modalities. Adopting the nomenclature utilized in [33, 40], we will refer to these experiments as (1) *clinical*, using only clinical features, (2) *image*, using 2.5D clinical images only, and (3) *combined*, using a combination of clinical and 2.5D clinical images. Each experiment was performed with and without the addition of IDF.

#### Clinical

The clinical experiments utilized the tabular EHR features, both those listed in Table 1 with the exception of scanner manufacture and field strength and those listed in Table A3. The classifier architecture was used, with a constant learning rate of $0.01$. Models were trained with just the EHR features, just the IDF features, and a combination of the EHR and IDF features.

#### Image

**Resnet**: Prior to training, the 2.5D DWI was first multiplied with the provided binary brain mask. Three types of Resnet experiments were performed: *transfer learning* (TL), *fine-tuning* (FT), and training from *scratch*. Both the TL and FT experiment started with a pretrained Resnet10 backbone previously trained on 23 2.5D medical image datasets [59]. During TL, the model weights in the pretrained backbone were frozen and only the classifier was trained using the same learning rate as the clinical experiments. During FT, the entire pretrained architecture was trained with the learning rate scheduler and an initial learning rate of $0.001$ following the procedure outlined in [33, 59]. To train the model from scratch, the network was initialized with random weights and trained with the scheduler and an initial learning rate of $0.1$ [59]. The fine tuning and scratch experiments were also performed with the addition of a squeeze and excite (SE) layer [60] following the last residual layer as outlined in Appendix B and Fig. 2. Finally, the best performing model from all of these experiments on the validation data was selected. The model weights for all but the classifier were loaded and frozen and the classifier was trained with the addition of the IDF.

**Autoencoder**: The autoencoder was trained using the 2.5D DWI images as both the input and the output of the model. Additional training details are provided in Appendix C.2. After training, the resulting encoder was employed to generate embeddings (ie latent vectors) for the 2.5D DWI from all the data sets. The training embeddings were used to train (and evaluate) a subsequent classifier using the classifier architecture described in B.1 and the same setup as the clinical experiment above both with and without IDF.

#### Combined

In the combination experiments, the trained Resnet and autoencoder from the image experiment were utilized. Only the classifier (Appendix B.1) was re-trained (experimental setup equivalent to Sect. “Clinical”) for each, with the addition of the EHR (and IDF) features.

## Results

### LOS

**Fig. 3:**
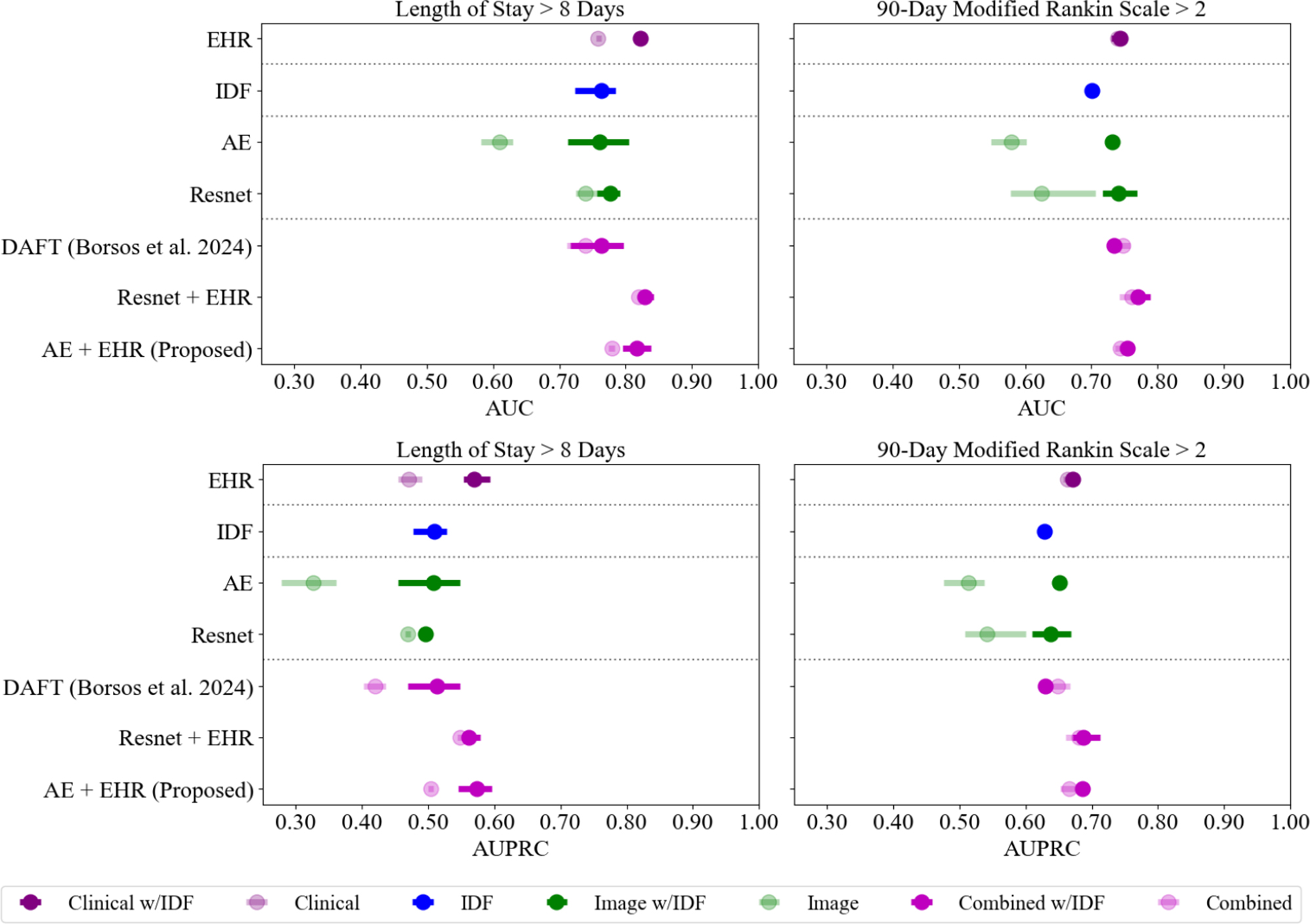
Comparison of AUC and AUPRC test performance averaged across folds for each outcomes across all methods tested. *AUC: area under the receiver operating characteristic curve; AUPRC: area under the precision recall curve; EHR: electronic health records; IDF: image-derived features; AE: autoencoder; DAFT: dynamic affine feature map transform*

In the LOS cohort there were $$1491$$ AIS patients with an average age of $$62.28$$. Of those, $$282$$ ($$18.91\%$$) had a LOS $$> $$ 8 days. A comparison of the area under the receiver operating characteristic curve (AUC) and AUPRC across experiments is visualized in Fig. 3 and all metrics are presented in Table 2. The clinical experiment using only EHR values resulted in an average AUPRC of 0.470 and an F1-Score of 0.469 when evaluating on the hold out test set. Adding IDF to the EHR increases the average performance by 0.099 and 0.071 for AUPRC and F1-Score respectively. This EHR and IDF model out-performs the model using IDF features alone (Fig. 3).

**Table 2:** Average (standard deviation) of performance metrics across folds on hold out test set for all experiments predicting length of stay (LOS) $>$ 8 days

		AUC	AUPRC	Recall	Specificity	F1-Score
*Clinical*	EHR	0.758 (0.004)	0.470 (0.015)	0.579 (0.014)	0.791 (0.017)	0.469 (0.022)
	EHR w/IDF	0.822 (0.002)	0.569 (0.018)	0.672 (0.065)	0.809 (0.029)	0.540 (0.015)
	IDF	0.763 (0.028)	0.509 (0.023)	0.462 (0.033)	0.889 (0.015)	0.477 (0.011)
*Image*	Resnet	0.739 (0.020)	0.470 (0.003)	0.427 (0.054)	0.863 (0.029)	0.423 (0.019)
	Resnet w/IDF	0.776 (0.015)	0.496 (0.007)	0.415 (0.060)	0.900 (0.030)	0.449 (0.022)
	AE	0.610 (0.020)	0.326 (0.035)	0.444 (0.016)	0.760 (0.021)	0.361 (0.011)
	AE w/IDF	0.761 (0.038)	0.508 (0.039)	0.485 (0.022)	0.873 (0.013)	0.480 (0.029)
*Combined*	DAFT [32]	0.739 (0.020)	0.420 (0.014)	0.719 (0.014)	0.664 (0.024)	0.457 (0.022)
	DAFT w/IDF	0.764 (0.034)	0.514 (0.033)	0.684 (0.029)	0.735 (0.029)	0.487 (0.023)
	Resnet	0.820 (0.001)	0.548 (0.005)	0.579 (0.057)	0.849 (0.032)	0.521 (0.007)
	Resnet w/IDF	0.829 (0.009)	0.562 (0.012)	0.602 (0.079)	0.847 (0.032)	0.532 (0.027)
	AE	0.779 (0.004)	0.504 (0.003)	0.644 (0.016)	0.769 (0.012)	0.490 (0.016)
	AE w/IDF	0.817 (0.018)	0.573 (0.021)	0.667 (0.066)	0.825 (0.014)	0.552 (0.028)

AUC: area under the receiver operator curve; AUPRC: area under the precision recall curve; EHR: electronic health records; IDF: image-derived features; AE: autoencoder; DAFT: dynamic affine feature map transform

The validation and test results for all Resnet image experiments are presented in Appendix D. The best performing model on the validation data was the FT approach with an AUPRC of 0.436 (Table D4). This fine-tuned Resnet was selected for comparison with the other experiments as shown in Table 2. It had a AUPRC of 0.470 and an F1-Score of 0.423 when applied to the hold out test set (Table D5). When IDF features were added, the AUPRC performance increased by 0.026 but it still did not out-perform the IDF model from the clinical experiments. The autoencoder embeddings by themselves did not out-perform the Resnet, however when IDF were added, it out-performed all other experiments and models with an average test AUPRC of 0.573 and an F1-Score of 0.552 (Table 2). Finally we compared to the DAFT model proposed in [32] as a light-weight data fusion alternative for tabular and imaging data; this model performed performed worse than using EHR alone in this dataset.

### 90-day mRS

In the 90-day mRS cohort, $375$ out of $974$ patients ($38.50\%$) had a 90-day mRS $>$ 2. The clinical experiment resulted in average test AUPRCs of 0.628, 0.664, 0.672 for IDF, EHR, and EHR with IDF respectively (Fig. 3). Adding IDF to EHR did not tangibly improve the average performance (Table 3).

**Table 3:** Average (standard deviation) of performance metrics across folds on hold out test set for all experiments predicting 90-day Modified Rankin Scale (mRS) > 2

		AUC	AUPRC	Recall	Specificity	F1- Score
*Clinical*	EHR	0.739 (0.007)	0.664 (0.006)	0.644 (0.023)	0.689 (0.063)	0.602 (0.019)
	EHR w/IDF	0.743 (0.009)	0.672 (0.007)	0.644 (0.012)	0.719 (0.019)	0.616 (0.009)
	IDF	0.701 (0.004)	0.628 (0.002)	0.578 (0.041)	0.697 (0.048)	0.560 (0.011)
*Image*	Resnet	0.625 (0.058)	0.542 (0.041)	0.676 (0.148)	0.452 (0.218)	0.525 (0.015)
	Resnet w/IDF	0.741 (0.021)	0.637 (0.024)	0.658 (0.045)	0.700 (0.010)	0.614 (0.024)
	AE	0.580 (0.022)	0.513 (0.027)	0.796 (0.098)	0.261 (0.168)	0.534 (0.018)
	AE w/IDF	0.731 (0.004)	0.651 (0.008)	0.649 (0.028)	0.675 (0.031)	0.598 (0.006)
*Combined*	DAFT [32]	0.747 (0.008)	0.649 (0.013)	0.720 (0.011)	0.619 (0.028)	0.619 (0.016)
	DAFT w/IDF	0.735 (0.002)	0.630 (0.006)	0.689 (0.028)	0.708 (0.018)	0.639 (0.011)
	Resnet	0.761 (0.016)	0.680 (0.017)	0.711 (0.050)	0.700 (0.024)	0.648 (0.021)
	Resnet w/IDF	0.770 (0.014)	0.687 (0.018)	0.707 (0.047)	0.725 (0.041)	0.658 (0.021)
	AE	0.744 (0.009)	0.666 (0.010)	0.685 (0.017)	0.689 (0.035)	0.628 (0.010)
	AE w/IDF	0.754 (0.004)	0.685 (0.006)	0.627 (0.022)	0.767 (0.013)	0.626 (0.013)

AUC: area under the receiver operator curve; AUPRC: area under the precision recall curve; EHR: electronic health records; IDF: image-derived features; DAFT: dynamic affine feature map transform

The validation and test results for the Resnet image experiments are presented in Tables E6 and E7 respectively in Appendix E. The Resnet model trained from scratch with the squeeze and excite (SE) module performed the best on the validation set with an average AUPRC of 0.586 and an F1-Score of 0.531 (Table E6). The FT model performed similarly but with a larger standard deviation. Therefore, the Resnet model trained from scratch with the SE module is presented in Table 3 in comparison to the other experiments with an average test AUPRC of 0.542 and F1-Score of 0.452. When IDF were added to this model, the performance increased to 0.637 and 0.614 for AUPRC and F1-Score respectively. The autoencoder for the imaging experiment had slightly inferior performance without IDF compared to this Resnet, 0.513 AUPRC and 0.534 F1-Score, but superior performance when IDF were added, 0.651 AUPRC and 0.598 F1-Score (Table 3).

As shown in Table 3, taking the best Resnet model mentioned above, the classifier was retrained with the addition of EHR data to achieve an average test performance of 0.680 AUPRC and an F1-Score of 0.648. Adding IDF features to this model increased the AUPRC performance by 0.007 and the F1-Score by 0.01 without having significant impact on the standard deviation (Table 3). The autoencoder with EHR and IDF in the combined experiment performed similarly to the Resnet with EHR and IDF but with a smaller standard deviation (Fig. 3).

## Discussion

The highest performance in terms of mean test AUPRC across folds was obtained with the autoencoder method combined with IDF and clinical data for LOS $>$ 8 days (AUC: 0.817; AUPRC: 0.573; Recall: 0.667; Specificity: 0.825; F1-Score: 0.552)(2). For 90-day mRS $>$ 2, the highest mean test AUPRC was achieved with the Resnet backbone, IDF, and clinical data, however the autoencoder method with the same auxiliary features had similar performance with smaller standard deviations (AUC: 0.754; AUPRC: 0.685; Recall: 0.627; Specificity: 0.767; F1-Score: 0.626)(3). A detailed sensitivity analysis is presented in Appendix G.

In predicting LOS $>$ 8 days, IDF improved average AUPRC performance in all but the Resnet combined experiment. However, in predicting 90-day mRS $>$ 2, IDF only improved the average image experiment performances and reduced the standard deviations without significantly improving the EHR or combined experiments. Lesion volume measured from DWI has been shown to decrease in the 24 hours following early perfusion suggesting that imaging and IDF are more relevant in predicting short-term outcomes [61]. Furthermore, lesion volume is significantly correlated with both outcomes, likely acting as a proxy for severity (Appendix G Fig. G3). However, the increases in the imaging experiments over the IDF model alone, particularly for mRS, suggest that there is additional information captured from the 2.5D DWI in addition to what is already curated in the IDF.

Grad-CAM images [62–64], which allow for visualization of which parts of the image impacted the prediction, were generated for the selected Resnet image experiments with the highest validation AUPRC. The folds with the best validation AUPRC were chosen to generate the maps and single slices from the volumes were randomly selected for visualization. Grad-CAM maps for LOS and mRS are presented in Figures F1 and F2 respectively. As shown in Fig. F1, the LOS model’s region of interest overlaps nicely with the provided stroke mask even when the lesion appears in different parts of the brain or in different sizes. Similarly, in Fig. F2, the mRS model is fairly good at identifying stroke regions. However, when the stroke lesion is small or non-existent as shown in sub-panel a, the model utilizes little information from the slice.

Few studies to date have utilized artificial intelligence to predict LOS for AIS patients. Feyen et al. utilized baseline admission data, periprocedural, and imaging data to predict LOS $>$ 10.7 days (the mean LOS in their cohort) for AIS patients treated with thrombectomy utilizing a fairly small samples size (*N*=$113$) [10]. Their random forest model achieved an AUC of 0.73, specificity of 0.68, and sensitivity of 0.80 [10]. Bacchi et al. trained a model to predict LOS $>$ 8 days for AIS and intracerebral hemorrhage patients (*N*=$2840$) using six admission variables selected during feature selection (ability to walk at time of admission, result of initial swallowing screening, pre-stroke mRS, age at time of admission, National Institutes of Health Stroke Scale (NIHSS) at time of admission and socioeconomic status) achieving an AUC, recall, specificity, and F1-Score with linear regression of 0.66, 0.64, 0.62 and 0.72 respectively [6] with only a slight drop when applying to prospective or external datasets [5]. The proposed autoencoder model is better overall at distinguishing between the classes and identifying true negative cases than both of these previous studies, but it is less precise. Given the inherent complexity of hospitalizations, utilizing admission data to predict long LOS can be complicated by factors such as potential surgical complications, hospital acquired infections, and discharge care availability. Therefore, the operational impact of such a model likely lies in the specificity, predicting short LOS with the absence of any non-stroke related complications. Our model’s higher mean specificity over previous models shows provides support for future implementation.

Unlike LOS, 90-day functional outcomes measured by mRS have been widely studied using AI methods in stroke patients. The majority of studies utilized the same dichotomization threshold: 90-day mRS $>$ 2 although the directionality for assignment of the positive class varies. Studies such as [53] utilized clinical variables at admission for AIS patients receiving specific treatments with the best performing model achieving an AUC of 0.83 [53]. Ramos et al. performed two experiments to fuse clinical features with CTA images, first utilizing region-based radiomic features and second a 2.5D Resnet architecture with SE layers [33]. They found that adding the imaging data in either form did not increase the performance compared to a model trained on the clinical data alone which had an AUC and F1-Score of 0.81 and 0.71 respectively [33]. While our model improved when adding IDF (lesion volume and thrombus location), their IDF included features extracted from 70 atlas regions. In addition, CTA may not adequately capture the extent of infarction. Likewise, Mutke et al. extracted MRI-derived IDF which also did not improve their baseline clinical model (AUC 0.71) [39]. Borsos et al. recently proposed the DAFT model as a light-weight alternative to large CNN models with late fusion of clinical data; utilizing CT perfusion and clinical data they achieved an AUC and F1 score of 0.75 and 0.80 respectively [32]. With a relatively small dataset (*N*=$250$), Nishi et al. deployed a CNN for patients with large vessel occlusion (LVO) in anterior circulations receiving treatment showing improvement when using DWI over using the ASPECTS and ischemic core volume alone (0.73, 0.64 and 0.67 respectively) [51]. For the same type of patients, Sommer et al. found that incorporating treatment information with clinical data and CTA using a 2.5D pretrained Resnet50 resulted in increased performance (AUC of 0.86) [25]. Samak et al. performed a similar study for general acute AIS patients using non-contract CT images and clinical data resulting in an AUC of 0.75 and a F1-Score of 0.62 [26, 52]. While these studies have shown promise, few have focused specifically on post-intervention predictions, however, our results are on par with these previous results in terms of raw performance metrics of long-term functional outcomes for stroke patients. Unlike previous studies though, our approach did show a marginal increase in performance when adding IDF and imaging to the clinical features.

The proposed approach has numerous advantages over the traditional CNN-based image fusion. First, although the autoencoder model has more parameters overall (19k vs. 14k), it is a much simpler architecture (as illustrated in Fig. 2). While CNNs capture spatial features relevant to predicting a specific outcome, an autoencoder captures and compresses the most essential aspects of an image agnostic of any outcome which is considered a simpler task than categorizing data into predefined categories. This allows an autoencoder to be trained once per modality and utilized across outcomes with various combinations of auxiliary data, thus reducing the overall training time and computational resources required for development of models for multiple related classification tasks. Furthermore, this two-stage modular approach enables the combination of embeddings across sequences or imaging modalities to boost performance, a complicated task when utilizing CNNs.

In addition to the technical implications of this work, an equally important clinical component exists. Operationally within the healthcare system, it would be beneficial to predict how long the patient may need inpatient care to allocate appropriate resources and plan for post-discharge care. Our model’s performance in AUPRC and F1-Score, while still modest in part due to the complex nature of inpatient stays, illustrates a heightened ability to more accurately predict LOS than other methods specifically in an imbalanced dataset as is often the case when considering more extreme outcomes. In practice, this would translate to a balance between identifying patients with expected long LOS while having a relatively high specificity (ie low false positive rate) and correctly identifying patients with short LOS. Given the complexity of clinical care, false negatives are expected since the model in its current state is not designed to account for recovery trajectories due to specific interventions, incurred complications or infections, issues being treated concurrently, or delays in discharge due to lack of available follow-up care.

Likewise, for patients and their families, the post-intervention window of a stroke is often filled with uncertainty and clinicians lack sufficient data-driven guidance as to the patient’s probability and extent of recovery. Our 90-day mRS model had a higher mean specificity compared to all other modeling approaches which would translate clinically into better identifying patients at a low risk of long-term disability. However, relatively low sensitivity may result in the model predicting no long-term disability when it in fact the patient did experience a negative outcome. This model at its current state is designed to inform patients and their families about potential long-term outcomes, of which current guidance is minimal, and not directly drive clinical decisions. In practice, incorporation of additional recovery trajectory variables would be critical for more accurately assessing long-term disability prior to use in clinical decision support.

While numerous other attempts have been made to model outcomes, these have largely been performed with limited datasets and disregarding the intricacies of clinical implementation and generalization across healthcare systems. Specifically, relying on EHRs alone becomes complicated when different variables are collected across health systems, and data is missing particularly during emergency admission. In addition, implementing models into EHR systems often takes significant effort as each EHR company has its own standards, data formats, and deployment processes. Although imaging also has variations (scanners, resolutions, positioning etc.), if appropriately accounted for, imaging alone may allow for clinical prediction models that are easier to implement and generalize better across populations while maintaining similar levels of performance. While our results show this could be done with IDF alone, curating these variables takes manual effort from experts which are often not available for timely analysis, especially at rural institutions. The results of this study, while showing marginal improvement when adding IDF to the 2.5D MRI embeddings and EHR, suggest that with further modeling, it may be possible to rely on only reliable data sources without losing performance. Furthermore, it is possible that this approach could also remove some underlying bias by focusing on the anatomical structures instead of sparse and inconsistent EHR variables.

This research presents several limitations that could be addressed in future studies. Firstly, a significant limitation is the lack of external validation. Although AIS datasets exist, most do not include DWI, which is the foundation for the models proposed in this work. Future work will focus on validating this method against external datasets using alternative MRI sequences, as well as on internal datasets. Secondly, the proposed autoencoder method, while demonstrating comparable or modest improvements in predicting short-term outcomes, suffers from a lack of explainability and transparency compared to CNNs or standard machine learning techniques such as random forest. Enhancing model interpretability in subsequent work will be crucial for clinical adoption. Additionally, our current approach does not handle missing variables, a common issue in clinical data that could hinder this method’s integration and generalization into clinical workflows. Due to constraints on computational resources and time, we did not perform a exhaustive parameter tuning, which could potentially lead to marginal performance improvements for the models. Variables such as stroke onset time and treatment, although likely valuable in predicting outcomes [50, 65], were not included as a limitation of data availability in the utilized dataset. Lastly, our method utilizes MRI data from a single sequence at a single time point. Anatomical changes such as formation of edema may not show up on initial DWI but may impact long-term outcomes [66]. Expanding the model to incorporate multiple sequences or time points, when available, could significantly enhance predictive accuracy.

## Conclusion

This study presents a novel approach to predicting both short-term operational and long-term functional outcomes for patients with AIS through the integration of 2.5D MRI data and EHR using an autoencoder. Our findings indicate that the proposed method provides comparable predictive performance to traditional models. By enabling the fusion of complex 2.5D imaging with clinical data, this method offers a more comprehensive assessment of stroke outcomes than utilizing EHR or IDF alone, which can be pivotal in informing personalized treatment strategies and optimizing resource allocation in healthcare settings.

Moreover, the ability of this method to encapsulate imaging data into interpretable embeddings implies potential for easier integration into clinical workflows, overcoming some of the implementation challenges faced with more complex CNN architectures. Although our approach demonstrates significant promise, further refinement and validation are needed across diverse patient datasets and clinical environments to ensure its robustness and generalizability. Additionally, enhancing model interpretability remains crucial for clinical adoption, particularly to gain trust among healthcare professionals.

Overall, this research provides a foundation for future work aimed at refining predictive models for AIS outcomes, with the ultimate goal of improving clinical decision-making and patient care in stroke management. By bridging the gap between sophisticated imaging techniques and practical clinical application, the work moves us closer to achieving a more efficient and precise healthcare system for stroke patients.

## Electronic supplementary material

Below is the link to the electronic supplementary material.

Supplementary Material 1

## Data Availability

The ICPSR dataset is available online (https://www.icpsr.umich.edu/web/ICPSR/studies/38464/summary). A software package for model inference can be accessed via github (https://github.com/kayvanlabs/ai-is-stroke-outcomes-dwi) with the model executables available at https://tinyurl.com/mrn4nwrm. The University of Michigan’s Innovation Partnerships (UMIP) unit will handle potential charges/arrangements of the use of data by external entities, using such methods as material transfer agreements. Please contact UMIP (innovationpartnerships@umich.edu) for inquiries.

## Abbreviations

AIS: Acute Ischemic Stroke
ASPECTS: Alberta Stroke Program Early CT Score
AUC: Area under the receiver operating characteristic curve
AUPRC: Area under the precision recall curve
CNN: Convolutional neural networks
CT: Computed tomography
CTA: Computed Tomography Angiography
DAFT: Dynamic Affine Feature Map Transform
DSC-PWI: Dynamic susceptibility contrast perfusion-weighted imaging
DWI: Diffusion weighted imaging
EHR: Electronic health records
FT: Fine-tuning
GPU: Graphical processing units
ICPSR: Inter-University Consortium for Political and Social Research
IDF: Image derived features
LOS: Length of stay
LVO: Large vessel occlusion
MRI: Magnetic Resonance Image
MNI: Montreal Neurological Institute
mRS: Modified Rankin Scale
SE: Squeeze and excite
TL: Transfer learning
