# Supplementary material for: AI post-intervention operational and functional outcomes prediction in ischemic stroke patients using MRIs
Source: BMC Med Imaging. 2025 Aug 14;25:329. doi: 10.1186/s12880-025-01864-1 (PMC12355742; doi:10.1186/s12880-025-01864-1)
Supplement: Supplementary file 1 — Supplementary Material 1 [file 12880_2025_1864_MOESM1_ESM.pdf]

Appendix A

Table A1: Demographic, stroke, and imaging characteristics for all ischemic stroke patients with available length of stay (LOS). Values for Age, BMI, NIHSS Initial, and Lesion Volume are displayed as mean (standard deviations) while all other values are displayed as count (percentage). *LOS: Length of Stay; NIHSS: National Institutes of Health Stroke Scale total; BMI: Body Mass Index; IVtPA: Thrombolysis with Intravenous Tissue-type Plasminogen Activator*

	**LOS $>$ 8 Days** (N=282)	**LOS $\leq$ 8 Days** (N=1209)	**p**†
**Age** (years)	62.48 (14.76)	62.23 (13.89)	0.60
**Male**	144 (51.06%)	659 (54.51%)	0.33
**BMI** (kg/m^2)	28.57 (6.80)	29.44 (7.60)	0.15
**Race**			0.01*
Asian	9 (3.19%)	31 (2.56%)	
African American	136 (48.23%)	680 (56.24%)	
Caucasian	102 (36.17%)	410 (33.91%)	
Missing	35 (12.41%)	88 (7.28%)	
**NIHSS Initial**	12.71 (7.49)	4.75 (5.47)	0.00*
**IVtPA before scan**	179 (63.48%)	821 (67.91%)	0.18
**Lesion Volume** (cm^3)	66.86 (91.55)	15.47 (34.89)	0.00*
**Thrombus Location**			0.00*
Bilateral	67 (23.76%)	141 (11.66%)	
Left	103 (36.52%)	491 (40.61%)	
Right	102 (36.17%)	443 (36.64%)	
Missing	10 (3.55%)	134 (11.08%)	
**Scanner Manufacturer**			0.45
Siemens	269 (95.39%)	1162 (96.11%)	
GE	9 (3.19%)	39 (3.23%)	
Phillips	3 (1.06%)	4 (0.33%)	
Unknown	1 (0.35%)	4 (0.33%)	
**Field Strength** (T)			0.97
1.5	171 (60.64%)	737 (60.96%)	
3.0	111 (39.36%)	472 (39.04%)	

† p-values from Mann-Whitney U test for continuous variables and Chi-squared test for categorical variables.

Table A2: Demographic, stroke, and imaging characteristics for all ischemic stroke patients with available 90-day modified Rankin Scale (mRS). Values for Age, BMI, NIHSS Initial, and Lesion Volume are displayed as mean (standard deviations) while all other values are displayed as count (percentage). *mRS: Modified Rankin Scale; NIHSS: National Institutes of Health Stroke Scale total; BMI: Body Mass Index; IVtPA: Thrombolysis with Intravenous Tissue-type Plasminogen Activator*

	**90-day mRS** > 2 (N=375)	**90-day mRS** ≤ 2 (N=599)	**p**†
**Age** (years)	65.58 (14.14)	60.42 (12.95)	0.00*
**Male**	183 (48.80%)	357 (59.6%)	0.00*
**BMI** (kg/m^2)	29.07 (7.78)	29.9 (7.40)	0.08
**Race**			0.97
Asian	10 (2.67%)	18 (3.01%)	
African American	199 (53.07%)	327 (54.59%)	
Caucasian	127 (33.87%)	209 (34.89%)	
Missing	39 (10.40%)	45 (7.51%)	
**NIHSS Initial**	9.91 (7.76)	3.64 (4.57)	0.00*
**IVtPA before scan**	285 (76.00%)	444 (74.12%)	0.56
**Lesion Volume** (cm^3)	46.26 (76.88)	11.04 (24.34)	0.00*
**Thrombus Location**			0.00*
Bilateral	76 (20.27%)	46 (7.68%)	
Left	150 (40.00%)	251 (41.90%)	
Right	133 (35.47%)	231 (38.56%)	
Missing	16 (4.27%)	71 (11.85%)	
**Scanner Manufacturer**			0.31
Siemens	365 (97.33%)	582 (97.16%)	
GE	10 (2.67%)	12 (2.00%)	
Phillips	0 (0.00%)	3 (0.50%)	
Unknown	0 (0.00%)	2 (0.33%)	
**Field Strength** (T)			0.69
1.5	216 (57.60%)	354 (59.10%)	
3.0	159 (42.40%)	245 (40.90%)	

† p-values from Mann-Whitney U test for continuous variables and Chi-squared test for categorical variables.

Table A3: Additional variables used in the *Clinical* and *Combined* experiments. Labs and vitals utilized were the first value recorded upon admission and were represented as real valued measurements. Prior medical conditions and medications were one-hot encoded.

Labs/Vitals	Prior Medical Conditions	Prior Medications
Systolic Blood Pressure	Atrial Fibrillation	Anticholesterol
Diastolic Blood Pressure	Carotid Stenosis	Anticoagulation
Cholesterol	Chronic Renal Insufficiency	Antiglucose
Triglycerides	Coronary Disease	Antihypertensive
High-density Lipoprotein	Diabetes Mellitus	Antiplatelets
Low-density Lipoprotein	Dyslipidemia	
Hemoglobin	Family History of Stroke	
Glucose	Heart Failure	
Creatinine	Hormone Replacement	
Prothrombin	Hypertension	
	Migraine	
	Obesity	
	Sleep Apnea	
	Peripheral Vascular Disease	
	Previous Stroke	
	Prosthetic Heart Valve	
	Sickle Cell	
	Smoker	
	Vein Thrombosis	

Appendix B Architecture Details

B.1 Classifier

The classifier architecture comprises a single hidden layer containing 10 nodes, employs a dropout rate of 0.2 for regularization, and includes a single output node.

B.2 Residual Network

Residual Networks (Resnet) are convolutional neural networks (CNN) that are commonly employed for medical image tasks such as segmentation and classification. Although these networks are often employed for 2D problems (ie slice-wise), due to the nature of stroke pathology and the importance of incorporating volume and location, it is necessary to input the entire 2.5D volume into the network. As model complexity scales with input features and the amount of data available was limited, a Resnet10 backbone [66] was selected for this task as opposed to some of the larger varieties. As shown in Figure 2, the network consists of a convolution, batch normalization, rectifier (ReLU), and max pooling layer in sequence followed by 4 residual blocks.

During some experiments, a squeeze and excite (SE) module was added after the last residual layer [60] as it has been demonstrated to enhance Resnet performance[33]. This module consisted of the following layers: adaptive average pooling, linear, ReLU, linear, and sigmoid multiplied back to the input before the SE module.

Finally, to perform classification, an additional global average pooling layer was added [67] to reduce the output across channels and dropout at a rate of 0.5 was applied before being fed into the classifier architecture described in B.1.

B.3 Autoencoder

The autoencoder encoder architecture consisted of three convolution layers with 32, 64, and 128 channels respectively each followed by a ReLU layer. The latent space had 10 nodes. The decoder mirrored the encoder.

Appendix C Training Details

Early stopping for all models was defined to stop training if generalization loss > 1 for five consecutive epochs or there was no decrease in lowest validation loss, defined as $E(t) > 0.999 * E_{opt}(t)$ where $E_{opt}(t) = \min_{t' \leq t} E_{val}(t')$, in 15 epochs[68]. Maximum epochs was set to 500. Network weights from the epoch with the lowest validation loss were used for inference.

All experiments were conducted using Python 3.12.4 utilizing PyTorch[69] and scikit-learn[57] as the machine learning frameworks. Training was performed on an internal virtual server at the University of Michigan with available graphical processing units (GPUs): 8 Nvidia TitanV GPUs with 12 GB VRAM and 3 Nvidia Tesla V100 with 16 GB VRAM.

C.1 Classifier and Resnet Implementation Details

The binary cross entropy (BCEWithLogitsLoss[57]) loss function was used in training. To account for class imbalance, a weight for the positive class (ie LOS > 8 days or mRS > 2) was incorporated into the loss function. The weight was calculated based on the training set $w_1 = \frac{n_0}{n_1}$ where n_0 and n_1 are the number of negative and positive samples respectively.

Stochastic gradient descent with a momentum of 0.9 and weight decay of 0.001 was adopted for all models. When specified, an exponential learning rate scheduler was used with a gamma of 0.95 updated every epoch was implemented.

A batch size of 10 was used in training models due to memory restrictions and multi-GPU data parallelization was implemented speed up training.

C.2 Autoencoder Implementation Details

Images were first min-max normalized. The autoencoders were trained using the Adam optimizer and an initial learning rate of 0.001 which was reduced by a factor of 0.1 after 2 epochs without a decrease in the validation binary cross entropy loss. A batch size of 2 was used for the autoencoder due to memory limitations.

Appendix D Length of Stay ResNet Results

Table D4: Average (standard deviation) of performance metrics across folds on respective validation sets for all Resnet-based image experiments predicting length of stay (LOS) $>$ 8 days. *AUC: area under the receiver operator curve; AUPRC: area under the precision recall curve; TL: transfer learning; FT: fine tuning; SE: squeeze and excite layer*

	**AUC**	**AUPRC**	**Recall**	**Specificity**	**F1-Score**
TL	0.626 (0.030)	0.359 (0.034)	0.244 (0.018)	0.904 (0.039)	0.297 (0.029)
FT	0.697 (0.018)	0.436 (0.039)	0.445 (0.065)	0.845 (0.016)	0.419 (0.053)
FT SE	0.691 (0.016)	0.428 (0.039)	0.430 (0.069)	0.831 (0.026)	0.398 (0.064)
Scratch	0.677 (0.088)	0.371 (0.117)	0.674 (0.103)	0.621 (0.204)	0.424 (0.062)
Scratch SE	0.711 (0.044)	0.406 (0.045)	0.644 (0.110)	0.667 (0.174)	0.432 (0.054)

Table D5: Average (standard deviation) of performance metrics across folds on hold out test set for all Resnet-based image experiments predicting length of stay (LOS) $>$ 8 days. *AUC: area under the receiver operator curve; AUPRC: area under the precision recall curve; TL: transfer learning; FT: fine tuning; SE: squeeze and excite layer*

	**AUC**	**AUPRC**	**Recall**	**Specificity**	**F1-Score**
TL	0.609 (0.013)	0.394 (0.014)	0.246 (0.062)	0.922 (0.041)	0.307 (0.030)
FT	0.739 (0.020)	0.470 (0.003)	0.427 (0.054)	0.863 (0.029)	0.423 (0.019)
FT SE	0.725 (0.028)	0.463 (0.008)	0.438 (0.052)	0.852 (0.037)	0.423 (0.012)
Scratch	0.654 (0.096)	0.370 (0.124)	0.626 (0.107)	0.600 (0.229)	0.397 (0.069)
Scratch SE	0.695 (0.035)	0.429 (0.009)	0.579 (0.124)	0.641 (0.195)	0.385 (0.048)

Appendix E Modified Rankin Scale ResNet Results

Table E6: Average (standard deviation) of performance metrics across folds on respective validation sets for all Resnet-based image experiments predicting 90-day Modified Rankin Scale (mRS) > 2. *AUC: area under the receiver operator curve; AUPRC: area under the precision recall curve; TL: transfer learning; FT: fine tuning; SE: squeeze and excite layer*

	**AUC**	**AUPRC**	**Recall**	**Specificity**	**F1-Score**
TL	0.531 (0.021)	0.446 (0.034)	1.000 (0.000)	0.003 (0.005)	0.557 (0.001)
FT	0.669 (0.096)	0.585 (0.096)	0.622 (0.109)	0.618 (0.034)	0.555 (0.085)
FT SE	0.655 (0.081)	0.576 (0.077)	0.650 (0.121)	0.552 (0.052)	0.546 (0.070)
Scratch	0.614 (0.045)	0.528 (0.051)	0.800 (0.062)	0.302 (0.044)	0.548 (0.031)
Scratch SE	0.649 (0.035)	0.586 (0.045)	0.672 (0.193)	0.490 (0.215)	0.531 (0.057)

Table E7: Average (standard deviation) of performance metrics across folds on hold out test set for all Resnet-based image experiments predicting 90-day Modified Rankin Scale (mRS) > 2. *AUC: area under the receiver operator curve; AUPRC: area under the precision recall curve; TL: transfer learning; FT: fine tuning; SE: squeeze and excite layer*

	**AUC**	**AUPRC**	**Recall**	**Specificity**	**F1-Score**
TL	0.492 (0.031)	0.413 (0.041)	1.000 (0.000)	0.003 (0.004)	0.555 (0.001)
FT	0.686 (0.054)	0.588 (0.049)	0.618 (0.049)	0.628 (0.031)	0.557 (0.037)
FT SE	0.657 (0.027)	0.563 (0.025)	0.658 (0.035)	0.551 (0.088)	0.553 (0.012)
Scratch	0.545 (0.045)	0.482 (0.037)	0.725 (0.088)	0.287 (0.034)	0.502 (0.042)
Scratch SE	0.625 (0.058)	0.542 (0.041)	0.676 (0.148)	0.452 (0.218)	0.525 (0.015)

Appendix F ResNet Grad-CAM

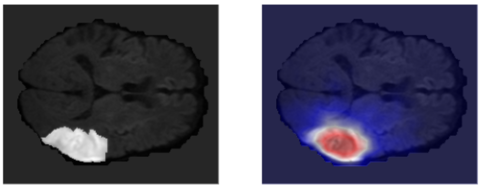

(a) False, Predicted Probability: 0.466

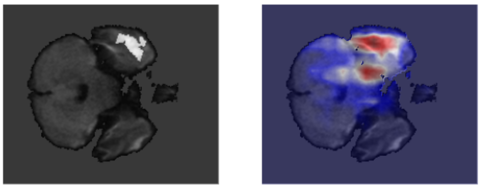

(b) False, Predicted Probability: 0.429

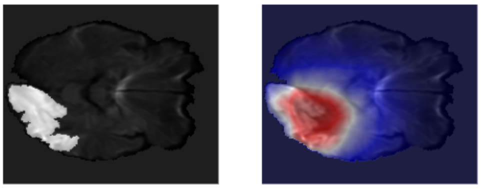

(c) True, Predicted Probability: 0.853

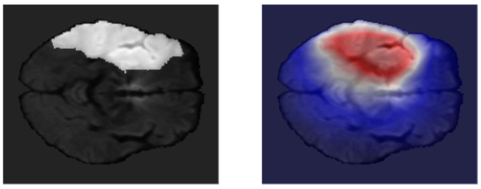

(d) True, Predicted Probability: 0.901

Fig. F1: Four randomly selected slices with overlaid stroke masks (left) and Grad-CAM maps generated from the image experiment with the pretrained Resnet10 fine-tuned to predict length of stay (LOS) $>$ 8 (right). Both the true class and predicted probability prior to thresholding at 0.5 are presented.

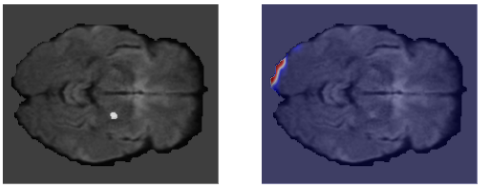

(a) False, Predicted Probability: 0.183

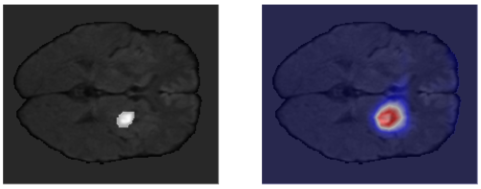

(b) False, Predicted Probability: 0.398

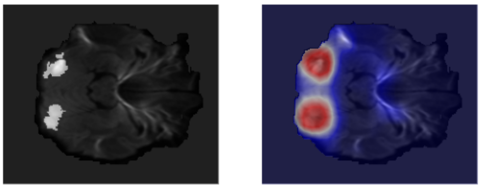

(c) True, Predicted Probability: 0.574

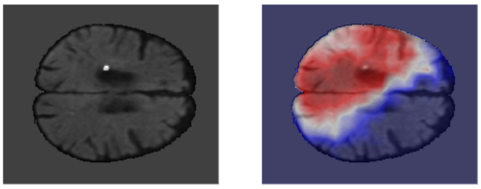

(d) True, Predicted Probability: 0.663

Fig. F2: Four randomly selected slices with the stroke mask overlaid (left) and Grad-CAM maps generated from the image experiment with Resnet10 trained from scratch with a squeeze and excite (SE) layer to predict 90-day Modified Rankin Scale (mRS) > 2 (right). Both the true class and predicted probability prior to thresholding at 0.5 are presented.

Appendix G Sensitivity Analysis

To assess the relatively low recall across all models, the statistical differences between the true positive and false negative patients (ie patients with LOS > 8 days or 90-day mRS > 2 that were correctly or incorrectly predicted by the models respectively) in the test set across each fold were analyzed. Categorical and binary variables were assessed with a Chi squared test and numerical values with a Mann U Whitney test as this doesn't require equal samples sizes. The significance threshold was set at $p < 0.05$.

For patients with LOS > 8 days, the model misclassified patients (ie predicted LOS $\leq$ 8 days) at a statistically significant rate for patients who did not have a history of diabetes or previous use of anti-glucose medications (2 folds: $\chi^2(1, N = 57) = 5.235, p = 0.022$; $\chi^2(1, N = 57) = 4.744, p = 0.029$), who had a low lesion volume (2 folds: $z = 441.000, p = 0.023$; $z = 490.000, p = 0.017$), were younger (1 fold: $z = 506.500, p = 0.008$), and who had not previously been on antiplatelets (1 fold: $\chi^2(1, N = 57) = 4.506, p = 0.034$). In addition, history of hypertension was statistically different in one fold among LOS > 8 days patients, ($\chi^2(1, N = 57) = 3.959, p = 0.045$). Of the patients predicted to have LOS $\leq$ 8 days (ie false negatives), 53% did not and 47% did have a history of hypertension. Likewise, of the true positives, 21% did not and 78% did have a history of hypertension respectively. Therefore, we can conclude this model was better at correctly classifying patients with LOS > 8 days when they had a history of hypertension. While these errors could be artifacts of the test size limited by the sample size of this dataset, they also provide insight into features that may define sub-phenotypes requiring additional modeling. Specifically, the models may be using age and medical history such as diabetes, hypertension, and antiplatelets as proxies for medical complexity which is assumed to be associated with longer LOS. Likewise, as shown in Figure G3 lesion volume is significantly correlated with LOS ($r = 0.233, p = 1.184e^{-08}$) likely acting as a proxy for severity.

For patients with 90-day mRS > 2, the model misclassified patients at a statistically significant rate who were male (2 folds: $\chi^2(1, N = 75) = 5.830, p - 0.016$; $\chi^2(1, N = 75) = 7.325, p = 0.007$), had a smaller lesion volume (3 folds: z=995.500, p < 0.001; z-995.500, p < 0.001; $z = 1111.000, p < 0.001$), and had a lower level of disability at 90-day (3 folds: $z = 915.000, p = 0.001$; z = $903.500, p = 0.005$; $939.000, p = 0.003$) measured by mRS. Lesion volume is significantly correlated with mRS ($r = 0.406, p = 3.83e^{-09}$) suggesting the models are using this as a proxy for stroke severity (Figure G3). The true positive patients had a mean 90-day mRS of 4.449 $\pm$ 1.209 while the false negatives had a mean 90-day mRS of 3.538 $\pm$ 0.905. Since mRS is a 7 point scale with scores of 2, 3, and 4 representing slight disability, moderate disability and moderately severe disability respectively, it is likely that imposing a hard threshold between 2 and 3 is an oversimplified threshold as there is likely some overlap between each subsequent score dependent on variations in individual's assessment and reporting of disability. As presented in Table G8, the models misclassify patients closer to the dichotomization threshold at a higher rate. For example, of the false negatives, 9.744% had a 90-day mRS of 3 while a total of 5.641% had a 90-day mRS > 3.

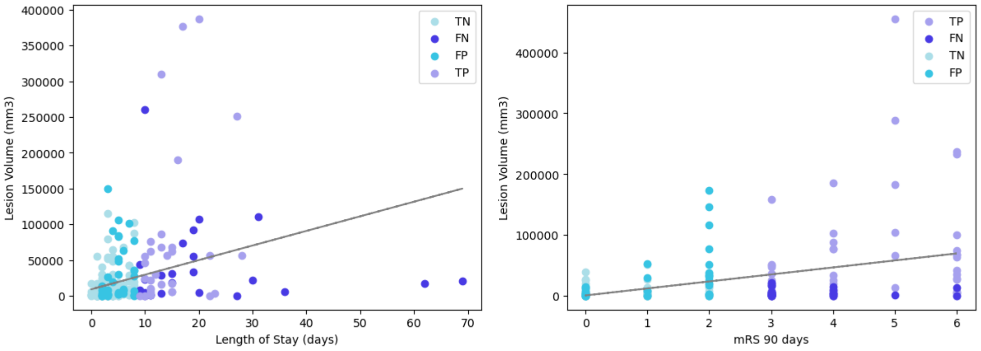

(a) LOS > 8 days (b) mRS > 2

Fig. G3: Correlation between outcomes and lesion volume in the test set. Coloring of confusion matrix presented for a single fold. *LOS: length of stay; mRS: modified Rankin scale*

Table G8: Confusion matrix representing average percentages of patients across all three folds, delineated by 90-day mRS before dichotomization. *mRS: Modified Rankin Scale*

**True Value**		**Predicted Value**	
		$mRS \leq 2$	$mRS > 2$
$mRS \leq 2$	0	25.128	4.103
	1	15.384	2.564
	2	7.692	6.667
$mRS > 2$	3	9.744	6.154
	4	3.077	7.179
	5	0.513	3.077
	6	2.051	6.667
